# A protocol to isolate and qualify purified human preantral follicles in cases of acute leukemia, for future clinical applications

**DOI:** 10.1186/s13048-017-0376-6

**Published:** 2018-01-05

**Authors:** Elodie Mouloungui, Tristan Zver, Christophe Roux, Clotilde Amiot

**Affiliations:** 1 University Bourgogne Franche-Comté, INSERM, EFS BFC, UMR1098, Interactions Hôte-Greffon-Tumeur/Ingénierie Cellulaire et Génique, F-25000 Besançon, France; 2 INSERM CIC-1431, University Hospital of Besançon, Clinical Investigation Center in Biotherapy, F-25000 Besançon, France; 3 Department of Reproductive Medicine and Biology, Cryobiology, University Hospital of Besançon, 3 boulevard Fleming, 25000 Besançon Cedex, France

**Keywords:** Collagenase NB6, Good manufacturing practices, Human follicle isolation, Leukemic cell purification

## Abstract

**Background:**

Autotransplantation of cryopreserved ovarian cortex can be associated with a risk of cancer cell reseeding. This issue could be eliminated by grafting isolated preantral follicles. Collagenase NB6 is an enzyme produced under good manufacturing practices (GMP) in compliance with requirements for tissue engineering and transplantation in humans and thus can be used to isolate preantral follicles from ovarian tissue in the framework of further clinical applications. Multicolor flow cytometry is an effective tool to evaluate the potential contamination of follicular suspensions by leukemic cells.

**Methods:**

The efficiency of collagenase NB6 was evaluated in comparison to collagenase type IA and Liberase DH, in terms of yield, morphology and viability. A short-term in vitro culture of follicles isolated with collagenase NB6 was conducted for 3 days in a fibrin matrix. A modelization procedure was carried out to detect the presence of leukemic cells in follicular suspensions using multicolor flow cytometry (MFC).

**Results:**

No statistical differences were found between collagenase NB6, Liberase DH (*p* = 0.386) and collagenase type IA (*p* = 0.171) regarding the number of human preantral follicles isolated. The mean diameter of isolated follicles was significantly lower with collagenase NB6 (*p* < 0.0001). The survival rate of isolated follicles was 93.4% (*n* = 272) using collagenase NB6 versus 94.9% (*n* = 198) with Liberase DH and 92.6% (*n* = 298) using collagenase type IA. Even after 3 days of in vitro culture in a fibrin scaffold, most of the isolated follicles were still alive after using collagenase NB6 (90.7% of viable follicles; *n* = 339). The rate of isolated Ki67-positive follicles was 29 ± 9.19% before culture and 45 ± 1.41% after 3 days. In 23 out of 24 follicular suspensions analyzed, the detection of leukemic cells by MFC was negative. The purification had no significant impact on follicle viability.

**Conclusion:**

The isolation and purification of human preantral follicles were performed following good manufacturing practices for cell therapy. Multicolor flow cytometry was able to confirm that final follicular suspensions were free from leukemic cells. This safe isolation technique using collagenase NB6 can be considered for future clinical applications.

## Background

Over the past 30 years, the survival rate of cancer patients has drastically improved thanks to early diagnosis, advances in surgery procedures and adjuvant therapies [1, 2]. Due to their toxicity, chemo- and radio-therapies can lead to side effects that affect the quality of life of those patients. Indeed, the risk of premature ovarian failure and infertility is important and varies depending on the age of the patient, type of drug, drug dose, protocol used, treatment duration and disease severity [3, 4]. For women diagnosed with cancer, desire to have a child is a major concern [5]. For this reason, techniques of fertility preservation have gained interest. Ovarian tissue cryopreservation is one of the available options to preserve fertility in women when the administration of a highly gonadotoxic treatment cannot be delayed and for prepubertal girls [6, 7]. Currently the re-use of frozen/thawed ovarian tissue, which can only be performed by autograft, has led to more than a hundred live births worldwide [7, 8].

However, in some cases of malignant disease, ovarian tissue transplantation is associated with a high risk of cancer reseeding [9–13]. The most common cancer in prepubertal girls is leukemia, with acute lymphoblastic leukemia (ALL) and acute myeloid leukemia (AML) accounting for 85 and 15% of cases, respectively [2]. Real time quantitative polymerase chain reaction (RT-qPCR) [10, 14], multicolor flow cytometry (MFC) [15–18] and ovarian tissue xenografts in immunodeficient mice [11, 19] are techniques proposed for the detection of minimal residual disease (MRD) in cryopreserved ovarian tissue.

Several alternatives to autograft of frozen/thawed ovarian tissue, such as the development of an engineered artificial ovary using mainly fibrin or alginate scaffolds [19–28], or in vitro folliculogenesis [29–39], were investigated based on the use of isolated preantral follicles, mostly primordial follicles. Primordial follicles are encapsulated in a basement membrane of collagen IV, laminin and to a lesser extent fibronectin [40]. Culturing isolated follicles separates them from surrounding stromal elements like capillaries, white blood cells and nerves and thus protects them from the risk of contamination with malignant cells, as cancerous cells are not located inside follicles but in the stroma [41].

In order to isolate follicles, techniques that consist of mechanical and/or enzymatic digestion of human cryopreserved ovarian tissue have been developed [37, 38, 42–45]. For clinical purposes, a highly-purified enzyme, free of endotoxins, must be used. Lierman et al., have compared the efficiency of Liberase DH, a purified good manufacturing practices (GMP) grade blend enzyme, to the first generation of Liberases (Liberase TM) and to collagenase IV [46]: these authors showed that Liberase DH allows isolation of viable primordial and primary follicles of higher quality and with good morphology and no sign of apoptosis, but the follicular yield was lower with Liberase DH than collagenase IV. Collagenases NB1 and NB6 are blends of GMP grade enzymes, which have been validated for their clinical applications; the highly purified collagenase NB1 has been used to isolate human pancreatic islets. When comparing the efficiency of Liberase DH and collagenase NB1, isolation yield and purity were found to be significantly higher with collagenase NB1, and in vitro function was well preserved [47]. Collagenase NB6, which is composed of collagenase type I and II, has also successfully been used in a clinical-grade protocol for the isolation of human umbilical cord tissue cells [48]. GMP grade collagenases NB1 and NB6 have shown their efficiency in different types of tissue but have not yet been tested for human ovarian follicle isolation.

Before considering the use of isolated human primordial follicles in patients with a risk of cancer reseeding, we have to ensure that the suspension containing isolated follicles is free of malignant cells. Soares et al., have developed a model of artificial addition of leukemic cell lines to suspensions obtained from ovarian cortical strip dissociation [42]. RT-qPCR was performed using the molecular marker BCR-ABL to detect the risk of ovarian residual disease. Even though few leukemic cells were retrieved during follicle pickup, they showed that three washes were sufficient to reduce the amount of leukemic cells detected in ovarian suspensions as the RT-qPCR was negative in all cases. In a recent study, Soares et al. found that 66% of malignant cells were present in cryopreserved ovarian tissue from 9 leukemia patients [49]. However, they confirmed by RT-qPCR analyses that three washes were able to eradicate malignant cells in follicular suspensions obtained from cryopreserved ovarian tissue of leukemia patients.

Multicolor flow cytometry (MFC), based on leukemic cell immunophenotype detection, is the only applicable technique when molecular markers are not available, and thus could be potentially applied to all leukemic patients [17]. MFC is able to detect one positive cell, but in hematology a significant abnormal cell population is defined as a homogeneous cluster of at least 20 LAIP+ (Leukemia-Associated ImmunoPhenotype positive) cells, referring to the common cut-off level used for the assessment of minimal residual disease in blood or bone marrow [50].

The aim of our study was first to evaluate the efficiency, in term of follicle yield, proliferation and viability, of an isolation technique using the highly purified GMP grade blend enzyme collagenase NB6, and second to ensure that the ovarian suspension containing isolated follicles is free of malignant cells after the initial addition of leukemic cells using MFC.

## Methods

### Collection of ovarian tissue

Ovarian biopsies (weight: mea*n* = 63.36 ± 30.49 mg; range = 13.9–171.2 mg; *n* = 43) were obtained from 16 women aged between 25 and 37 (mean age = 29.88 years) undergoing laparoscopic ovarian drilling for polycystic ovary syndrome. Human ovarian tissue obtained from laparoscopic drilling performed for treatment of women suffering from polycystic ovary syndrome has been used as reference tissue for our research for several years [51]. Ovarian cortical fragments were obtained using biopsy grasping forceps from an avascular portion of the ovary, before electrocoagulation of the puncture site. Specimens were then immediately transported to the laboratory in Leibovitz L-15® medium (Eurobio, France) on ice.

### Freezing/thawing of ovarian tissue

Cortical fragments were transferred in 1.8 ml cryovials (VWR, France) containing 1.5 ml of a freezing solution composed of 1.5 M dimethyl sulfoxide (DMSO; Sigma Aldrich, France) and 0.1 M sucrose (Pharmacy of Besançon University Hospital, France) in Leibovitz L-15® supplemented with 10% decomplemented AB human serum (Etablissement Francais du Sang, Bourgogne Franche-Comté, France). Frozen/thawed AB human serum was obtained from consenting male donors. Decomplementation was carried out by a 30-min incubation at 56 °C, and the decomplemented AB human serum obtained was then stored at 4 °C.

Cortical fragments in freezing solutions were incubated for 30 min on ice while gently rotated. Cortical biopsies were frozen according to a protocol using slow cooling with manual seeding in a programmable freezer (Planer® 360 or 560; Cryobiosystem, France) and stored in liquid nitrogen [51].

Cortical fragments were thawed according to a protocol previously described in the laboratory [52]. Briefly, the cryovials were warmed for 5 min in a heat chamber at 37 °C, ovarian tissue pieces were then washed in decreased solutions of DMSO 1.5 M (5 min), 1 M (5 min), 0.5 M (10 min) containing 0.05 M sucrose in Leibovitz L-15 supplemented with 10% decomplemented AB human serum. Ovarian tissue strips were finally transferred to a solution of Leibovitz L-15® supplemented with 20% of decomplemented AB human serum, for 10 more minutes.

### Ovarian tissue dissociation

This technique, previously published by our team [51], was modified as described below. Cortical fragments were cut into small pieces of ~ 1 mm^3^ and placed in a cryovial containing the enzymatic solution. They underwent mechanical and enzymatic dissociation using either collagenase IA routinely used in our laboratory (200 IU/ml final, Sigma Aldrich), a GMP grade purified collagenase NB6 (200 IU/ml final, Serva Electrophoresis GMbH; Coger, France), or Liberase DH (0.28 Wünsch U/ml, Roche, France).

Fragments dissociated with collagenase IA or collagenase NB6 were incubated for 3 h in a heat chamber at 37 °C and vortexed every 30 min. The collagenase activity was inhibited by the addition of 30% decomplemented AB human serum. The ovarian cell suspension obtained was filtered through a 60 μm nylon filter (Fisher Scientific, France).

The protocol for tissue dissociation with Liberase DH was a modification of the protocol described by Vanacker et al. [43]. Fragments dissociated with Liberase DH were incubated for 90 min in a heat chamber at 37 °C with gentle agitation. The collagenase activity was inhibited by the addition of 30% decomplemented AB human serum.

### Freezing/thawing of leukemic cells

Frozen/thawed blood or bone marrow (BM) cells originating from acute lymphoid leukemia (ALL) and acute myeloid leukemia (AML) patients (*n* = 8), with known LAIP, were used for serial dilutions of leukemic cells in ovarian cell suspensions obtained from healthy ovarian cortical strips. Blood or bone marrow cells were collected at diagnosis, frozen and stored in liquid nitrogen. Leukemic cells were thawed at 37 °C for 5 min, then added dropwise to a solution containing 10 ml of RPMI 1640 GlutaMax (Invitrogen, Cergy-Pontoise, France) supplemented with 20% fetal bovine serum (PAN Biotech GmbH, Aidenbach, Germany). The solution was then incubated for 15 min at 37 °C and centrifugated at 300 g for 7 min. Finally, the pellet was resuspended in RPMI 1640 GlutaMax supplemented with 20% fetal bovine serum for cell counting.

### Marking of leukemic cells using a cell tracer

Thawed leukemic cells were resuspended with 5 μM of CFDA SE Cell tracer (Vybrant® CFDA SE Cell Tracer Kit, Life Technologies SAS, France) as described by Soares et al., [42]. The CFDA SE Cell tracer allows for the labeling of leukemic cells in ovarian suspensions, with an inverted fluorescence microscope (CKX41, Olympus, France) by an intense green staining (ex/em: ~ 492/~ 517 nm). The observation of stained leukemic cells was performed punctually as a control of the isolation technique.

### Addition of leukemic cells in an ovarian cell suspension

An initial addition of leukemic cells was made to obtain a first ovarian cell suspension with an excess of either 10^6^ or 10^5^ leukemic cells, depending on the total leukemic cells available after count. From this first suspension, 1/10 dilutions were made twice to obtain decreasing concentrations of leukemic cells in ovarian cell suspensions containing isolated follicles (we thus obtained three ovarian cell suspensions containing either 10^6^, 10^5^, and 10^4^ leukemic cells or 10^5^, 10^4^ and 10^3^ leukemic cells). The amount of leukemic cells added to ovarian suspensions was defined so as to achieve a sensitivity to an order of 10^−5^,which is comparable to the threshold of sensitivity for minimal residual disease detection by multicolor flow cytometry [17]. The remaining solution of leukemic cells was used as positive control of the experiment.

### Follicle isolation and purification

The three resulting ovarian cell suspensions containing isolated follicles and leukemic cells were transferred to a Petri dish (IVF ICSI Nunc™ dish, 35 × 10 mm, Thermo Scientific, France) containing 100 μl of in vitro fertilization medium (IVF™, Vitrolife, France) under oil (Ovoil™, Vitrolife, France). Isolated follicles were then manually picked up using a denudation pipette with an inner diameter of 130 μm (Vitrolife, France) and transferred to an IVF 4-well cell culture plate (Nunc™, ThermoFisher Scientific, France), each well containing 30 μl of IVF medium under oil. The denudation pipette was changed after each dilution group was transferred. Follicles were washed 3 times in fresh IVF medium droplets, as described by Soares et al., [42]. The remaining droplet from the first pipetting of follicles (no wash) in the first well and the last drop containing washed isolated follicles in the fourth well (after 3 washes) were analyzed by MFC. Isolated follicles were manually counted using the Fertimorph™ vision software on a micromanipulation station (Research Instruments, France) under bright field, with a Hoffman modulation contrast objective (Nikon, France).

### Formation of a fibrin scaffold

The protocol used for the formation of a fibrin scaffold was a modification of the one previously described by Paulini et al. [53]. Evicel® is a fibrin sealant kit, routinely used in surgery and approved for clinical applications (Evicel®, Ethicon, France), which contains a solution of human fibrinogen (70 mg/ml) and a solution of human thrombin (1000 IU/ml). The stock solution of fibrinogen was diluted in alpha-minimum essential medium (αMEM, Sigma Aldrich, France) to obtain a final concentration of 50 mg/ml. Meanwhile, a stock solution of thrombin was also diluted in αMEM to obtain a final concentration of 10 IU/ml. A droplet of 15 μl of fibrinogen was placed in a 4-well cell plastic culture plate in which 30 to 100 preantral follicles isolated with GMP grade collagenase NB6 were carefully placed. No prior filtration step was carried out on ovarian suspensions from which isolated follicles would be embedded in fibrin matrices. The droplet containing isolated follicles was then mixed with 15 μl of thrombin solution. The resulting droplet of 30 μl was finally incubated 45 min at 37 °C to allow the fibrin to polymerize.

### In vitro culture of isolated follicles embedded in a fibrin scaffold

Fibrin scaffolds were submerged with ~ 200 μl of culture medium made up of αMEM supplemented with 10% decomplemented AB human serum, 10 μg/ml insulin, 5 μg/ml transferrin and 5 ng/ml sodium selenite (ITS supplement, Sigma Aldrich, France), and then incubated up to 3 days at 37 °C/5% CO_2_. Half of the medium was changed the second day of culture with fresh pre-warmed supplemented culture medium.

### Viability assessment

The initial health of isolated follicles was evaluated using trypan blue staining. Follicular suspensions were exposed to a 0.4% trypan blue solution (Sigma Aldrich, France) for 10 min. Dead follicles appeared in blue, and live ones were unstained after exposure to trypan blue staining [51].

Follicle viability was then assessed using a Live/Dead viability assay kit as described in the manufacturer’s protocol (Live/Dead® viability assay kit for mammalian cells, Life Technologies SAS, France). Briefly, isolated follicles were incubated in 30 μl of phosphate-buffered saline 1X (PBS; Cambrex, Belgique) containing 4 mM calcein AM and 2 mM ethidium homodimer-1 for 30 min at room temperature in the dark. After exposure to fluorescent dyes, follicles were observed under an inverted fluorescence microscope (CKX41, Olympus, France). Green fluorescence was visualized in live cells (ex/em: ~494/~517 nm) and red fluorescence in dead cells (ex/em: ~528/~617 nm). Pictures were taken with a Moticam Pro 282S camera (Motic, Hong Kong).

Isolated follicles were classified in four categories depending on the percentage of dead granulosa cells: V1, living follicles: follicles with the oocyte and all the granulosa cells viable (stained green); V2, minimally damaged follicles: follicles with less than 10% of granulosa cells dead (stained red); V3, moderately damaged follicles: follicles with 10–50% of granulosa cells dead; V4, dead follicles: follicles with the oocyte or over 50% of granulosa cells dead [54].

### Measurement of follicle diameter

Follicle diameters were calculated from measurements of isolated preantral follicles areas on images taken right after isolation and also after in vitro culture, using NIS software (NIS-Elements D 4.00.00, Nikon, France). Only follicles with the characteristic shape were considered. The basement membrane of follicles was taken as their outer limit. The follicle diameter was based on surface area measurement and computation of the theoretical diameter.

### Immunocytochemistry

Fibrin scaffolds (*n* = 4) were fixed in formol 4% for 24 h, dehydrated and embedded in paraffin. They were then cut with a rotary microtome (HM 340E, Microm Microtech, France) into 7 μm sections. Every five sections, slides were stained with hematoxylin-eosin (RAL Diagnostic, France) in order to confirm the presence of isolated follicles inside the gels. The proliferation of isolated human follicles seeded in fibrin clots was assessed by the presence of Ki67, which is localized in the nucleus and associated with cell proliferation. A rabbit anti-human Ki67 was used as the primary antibody (dilution 1/400, ref. RM-9106-S, ThermoFisher Scientific, France), and a mice anti-rabbit antibody from the same kit was used as a secondary antibody. Human fetal thymus obtained from our laboratory was used as a positive control, and the negative control consisted of buffer without primary antibody. The expression of Ki67 was characterized by a nuclear brown staining, and the slides were counterstained with anilin blue (ThermoFisher Scientific, France). Follicles were classified into two categories: non-growing follicles when all granulosa cells were Ki67-negative (blue coloration only), and growing follicles when at least one granulosa cell was Ki67-positive (brown coloration) [55].

### Multicolor flow cytometry (MFC) analysis

Markers of our experiments are routinely used in hematology for the diagnosis of leukemia. Eight-color MFC was performed using FACSCanto II™ flow cytometer and FACSDiva™ software version 6.1.3 (BD™ Biosciences, San Jose, CA, USA). The compensation matrix was set up using compbeads© (BD Biosciences) according to the manufacturer’s instructions. The same combinations of eight monoclonal antibodies were applied to ovarian cell suspensions and to different leukemic cell dilutions in ovarian cell suspensions.

MFC gating strategy was based on previous studies from our team [15–17]. Briefly, our gating strategy was based on the elimination of debris using forward (FSC) and side (SSC) light scatter characteristics. Nucleated viable cells were selected by their SYTO13^+^/7-AAD^−^ phenotype. Within these cells, we identified the CD45_low_ population, which corresponds to ovarian and leukemic cells.

The antibody panel included fixed monoclonal antibodies: 7-AAD (Beckman Coulter, France) and SYTO13 (Invitrogen, France) to gate the living nucleated cells as described below and CD45 V500 (Horizon 500, HI30; BD Biosciences) and CD3 V450 (Horizon 450; UCHT1; BD Biosciences) to characterize T lymphocytes (CD45^+^/CD3^+^). The antibody panel also included variable monoclonal antibodies expressed on leukemic cells at diagnosis (LAIP) coupled: with phycoerythrin (PE), against the antigens CD33 (P67.6; BD Biosciences), or CD44 (515; BD Biosciences), or CD58 (AICD58; Beckman Coulter), or CD361 (MEM-216; Exbio); with allophycocyanin (APC), against CD10 (HI10A; Beckman Coulter), or CLEC 12A (50C1; BD Biosciences), or CD117 (104D2; BD Biosciences); with allophycocyanin H7 (APC-H7), against CD43 (1G10; BD Biosciences), or HLA DR (G46–6; BD Biosciences); with phycoerythrin-cyanin 7 (PC7), against CD19 (J3.119; Beckman Coulter), or CD34 (581; Beckman Coulter); with V450 and Brillant Violet 421, against CD38 (HB7; BD Biosciences).

Antibodies were added to the cell suspension in 100 μl RPMI 1640 GlutaMax (Invitrogen, France) supplemented with 10% fetal bovine serum (PAN Biotech GmbH, Aidenbach, Germany) and incubated for 20 min at +4 °C. After being washed in PBS 1X, cells were resuspended in 100 μl of PBS 1X for MFC analysis.

A cell population was considered abnormal or positive when a homogeneous cluster of at least 20 events expressing the same LAIP as leukemic cells at diagnosis was detected [50]. When less than 20 events were detected by MFC, ovarian suspensions were considered negative or free of a significant number of malignant cells.

### Statistical analysis

Graphpad™ software was used for all statistical analysis. Student’s t test was used to compare the number of isolated follicles obtained after the dissociation with the GMP grade collagenase NB6, collagenase IA and Liberase DH. The Mann Whitney test was applied to compare the mean diameter of the follicles isolated from each enzyme used for ovarian tissue dissociation and to compare the mean diameter of isolated follicles after short-term in vitro culture. The comparison of the percentages of viable isolated follicles obtained with each enzyme was done using a chi-square test. Results were considered statistically significant when *p* ≤ 0.05.

## Results

### Evaluation of the GMP Collagenase NB6 for the isolation of human ovarian follicles

#### Follicle counting and classification

A total of 2674 follicles were isolated during our experiments (*n* = 31 cortical fragments). In order to validate our isolation technique, we first compared collagenase IA, which was previously used by our team, to collagenase NB6, which can be used in the clinic using the same concentration and incubation time. A total of 1361 (mean = 210 ± 205 for 100 mg of tissue; *n* = 13) follicles were isolated using the GMP grade collagenase NB6, and 1076 (mean = 127 ± 99 for 100 mg of tissue; *n* = 13) using collagenase IA. No significant difference was observed between the two enzymes concerning the number of follicles isolated (*p* = 0.171).

We then compared the follicles obtained after using collagenase NB6, collagenase IA and Liberase DH (Liberase DH has already been validated to isolate human follicles). A total of 532 (mean = 106 ± 97 for 100 mg of tissue; *n* = 5) follicles were isolated using the GMP grade collagenase NB6; 406 follicles (mean = 81 ± 68 for 100 mg of tissue; *n* = 5) were isolated with collagenase IA; and 327 follicles (mean = 65 ± 95 for 100 mg of tissue; *n* = 5) with Liberase DH. No significant differences were observed between the three enzymes concerning the number of follicles isolated (*p* = 0.386 for Liberase DH versus collagenase NB6; *p* = 0.720 for Liberase DH versus collagenase IA).

Isolated follicles were classified according to their stage for each isolation protocol. Most of the isolated follicles observed were primordial follicles no matter which enzyme was used: the rate of primordial follicles was 77.5% (*n* = 247/319) for collagenase IA, 83.7% (*n* = 303/362 isolated follicles) with collagenase NB6 and 75.1% (*n* = 154/205 isolated follicles) with Liberase DH. Isolation with Liberase DH yielded a higher rate of secondary follicles (10.3%; *n* = 21/205 isolated follicles) (Table 1).

**Table 1:** Classification of isolated follicles per protocol after enzymatic dissociation of ovarian cortical strips

	Follicles (n)	Primordial (%)	Primary (%)	Secondary (%)
Collagenase IA (200 IU/ml)	319	247 (77.5)	55 (17.2)	17 (5.3)
Collagenase NB6 (200 IU/ml)	362	303 (83.7)	48 (13.3)	11 (3)
Liberase DH (0.28 Wünsch U/ml)	205	154 (75.1)	30 (14.6)	21 (10.3)

% = percentage of each follicular stage out of the total isolated follicles per protocol

The mean diameter of follicles was evaluated on 781 isolated follicles. Their average diameter was significantly lower when they were isolated with collagenase NB6 (mean = 31.66 ± 6.79 μm; *n* = 317 follicles) when compared to collagenase IA (mean = 36.77 ± 7.69 μm; *n* = 273 follicles) (*p* < 0.0001), and also in comparison with Liberase DH (mean = 45.21 ± 8.26 μm; *n* = 191 follicles) (p < 0.0001).

#### Assessment of isolated follicle viability

Trypan blue staining was first used to assess the initial quality of isolated follicles. No significant differences were observed between trypan blue staining and the live/dead viability assay to determine the survival rate of isolated follicles (*p* = 0.99; *n* = 76 follicles analyzed).

After dissociation with collagenase NB6, 93.4% of the isolated follicles (*n* = 254/272 isolated follicles) were undamaged or minimally damaged (V1 + V2). The survival rate of follicles isolated with collagenase IA was 92.6% (*n* = 276/298 isolated follicles). After using Liberase DH, the percentage of viable isolated follicles was 94.9% (*n* = 188/198 isolated follicles) (Table 2).

**Table 2:** Viability of isolated follicles after dissociation of ovarian cortical strips

	Follicles (n)	V1	V2	V3	V4
Collagenase IA (200 IU/ml)	298	191	85	17	5
Collagenase NB6 (200 IU/ml)	272	194	60	14	4
Liberase DH (0.28 Wünsch U/ml)	198	145	43	10	0

Viability from V1 to V4, using collagenase IA (*n* = 7 patients), collagenase NB6 (*n* = 6 patients), and Liberase DH (*n* = 5 patients), according to the number of dead granulosa cells

**Fig. 1:**
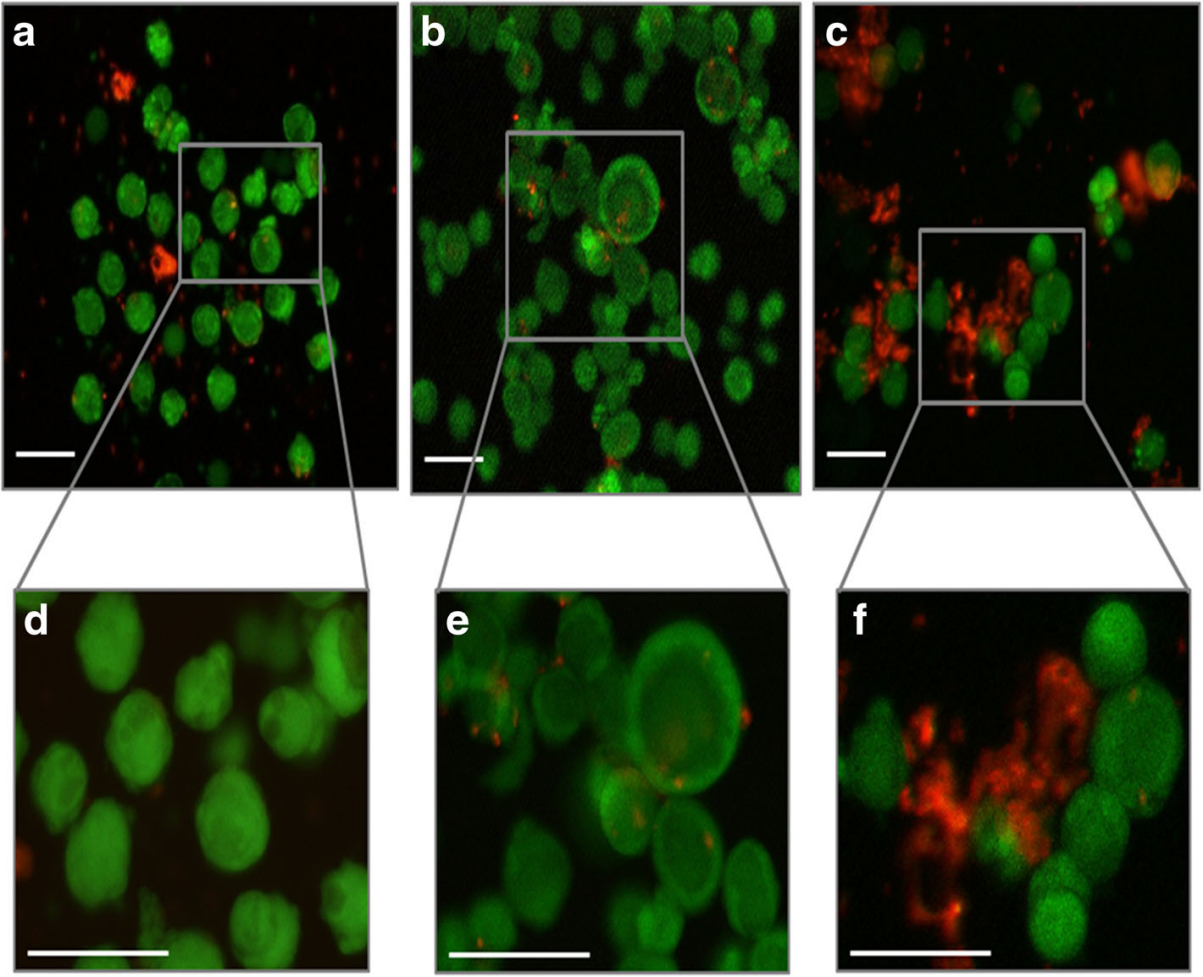
Viability assay performed on isolated follicles using the fluorescent probes calcein AM and ethidium homodimer-1. **a** and **d**. Isolated follicles after using collagenase NB6; **b** and **e**. Isolated follicles after using Liberase DH; **c** and **f**. Isolated follicles after using collagenase IA; green: live follicles; red: dead follicles; scale bar = 100 μm

There was no significant difference in terms of viability between the three isolation protocols (*p* = 0.959 for collagenase NB6 versus collagenase IA; *p* = 0.877 for collagenase NB6 versus Liberase DH; *p* = 0.728 for Liberase DH versus collagenase IA). However, more debris (stained in red by ethidium homodimer-1) were found when using collagenase IA (Fig. 1c and f).

### In vitro culture of human ovarian follicles isolated with GMP grade collagenase NB6

#### Follicle proliferation before and after in vitro culture in a fibrin scaffold

**Fig. 2:**
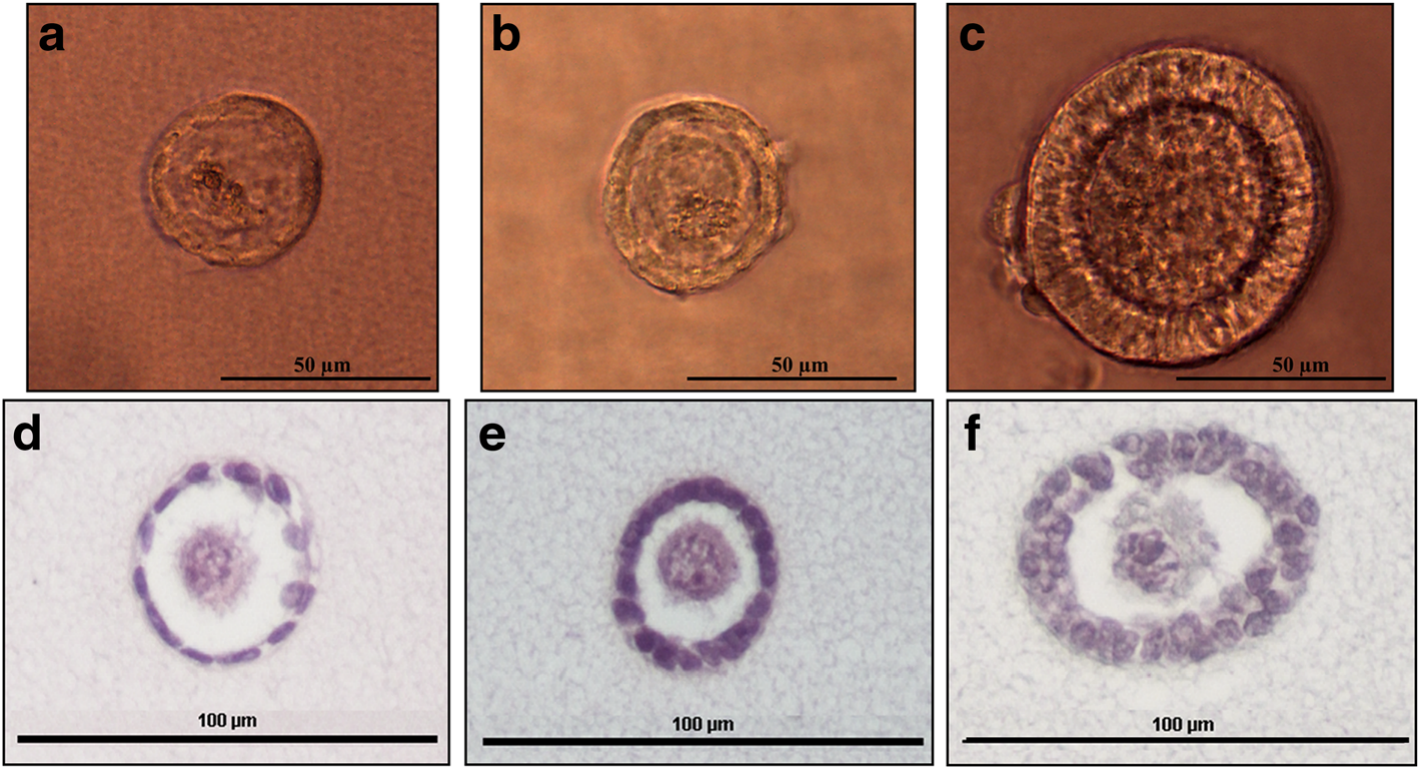
Isolated preantral follicles embedded in a fibrin scaffold. Brightfield images of an isolated primordial (**a**), primary (**b**) and secondary (**c**) follicle inside a fibrin scaffold; Hematoxylin and eosin staining of an isolated primordial (**d**), primary (**e**) and secondary (**f**) follicle inside a fibrin scaffold

The proliferative status of isolated preantral follicles was assessed after they were seeded inside a fibrin scaffold (Fig. 2). The mean rate of isolated follicles with Ki67-positive granulosa cells was 29 ± 9.19% (*n* = 9/31 follicles analyzed). Among the growing follicles, 23 ± 1.43% were small secondary follicles (*n* = 7/12 secondary follicles analyzed), and 6 ± 6.36% represented primary follicles (*n* = 2/13 primary follicles analyzed). No primordial follicles were Ki67-positive (*n* = 6 primordial follicles).

**Fig. 3:**
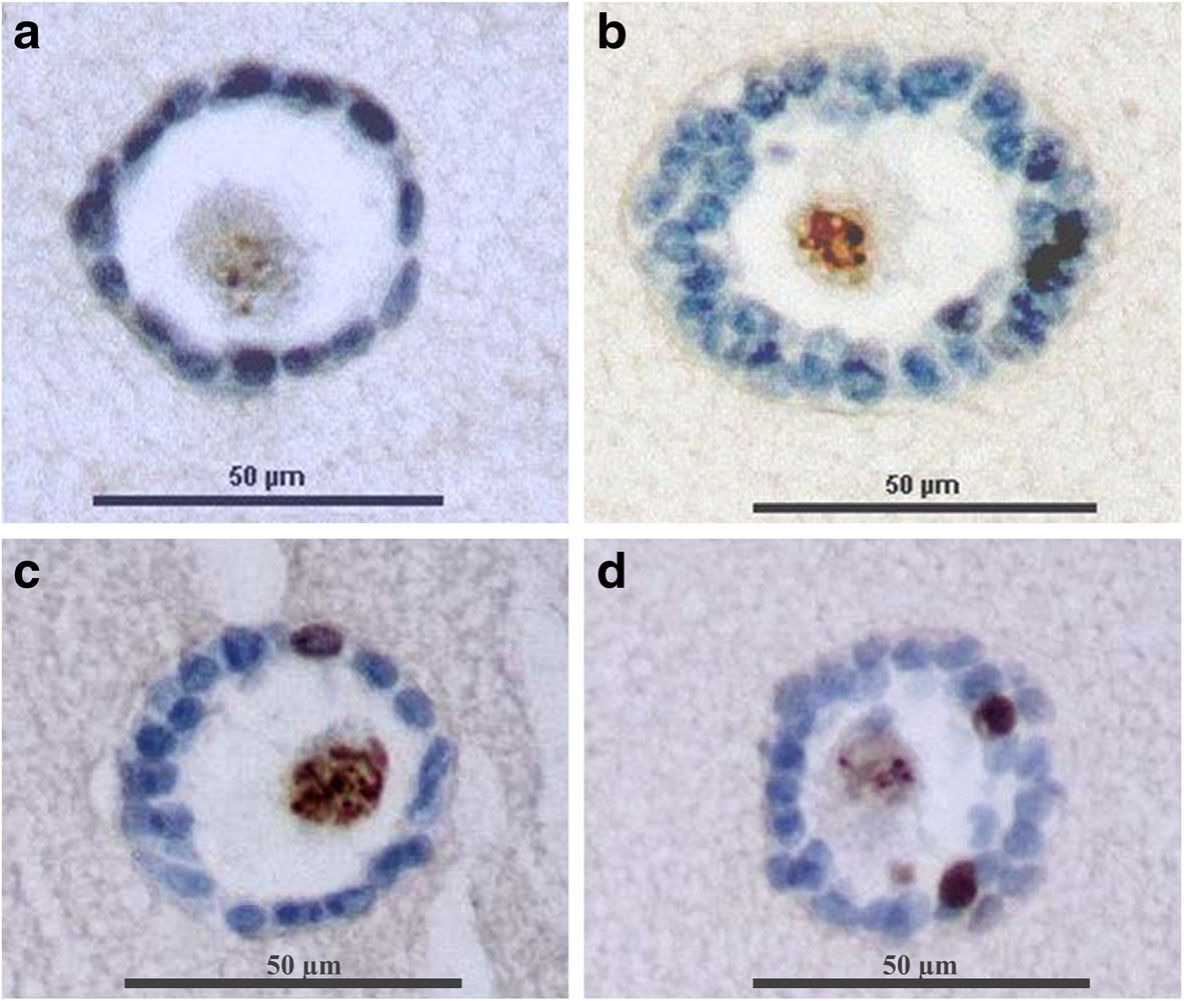
Immunohistochemistry of isolated preantral follicles embedded in a fibrin scaffold. **a** Embedded isolated primordial follicle with Ki67-negative granulosa cells in blue; **b** Embedded isolated secondary follicle with Ki67-negative granulosa cells in blue; **c** Embedded isolated primary follicles with one Ki67-positive granulosa cell in brown; **d** Embedded isolated secondary follicles with two Ki67-positive granulosa cells in brown

After 3 days of in vitro culture, 45 ± 1.41% of isolated follicles were Ki67-positive, or growing follicles (*n* = 10/22 follicles analyzed). Among them, 36 ± 2.83% were secondary follicles (*n* = 8/12 secondary follicles analyzed), 9 ± 1.41% were primary follicles (n = 2/6 primary follicles analyzed) and none of them were primordial follicles. All primordial follicles were Ki67-negative (*n* = 4 primordial follicles) (Fig. 3).

#### Assessment of isolated follicle viability

After 3 days of in vitro culture, the recovery rate of isolated follicles was 69%. Indeed, a total of 456 follicles (range: 36–136 follicles) were embedded in fibrin matrices after their isolation with collagenase NB6, whereas 293 embedded follicles (range: 11–81 follicles) were found after 3 days of short-term in vitro culture. The viability was assessed on fibrin scaffolds containing isolated follicles after 3 days of in vitro culture: we found that 90.7% of the isolated follicles (*n* = 307/339 follicles) were undamaged (76% of V1) or minimally damaged (15% of V2) (Table 3).

**Table 3:** Viability of isolated follicles in fibrin scaffolds after 3 days of in vitro culture

J3				
Number of fibrin gels (*n* = 9)	V1	V2	V3	V4
1	10	1	0	0
2	35	5	2	1
3	25	7	5	2
4	24	5	3	2
5	18	11	3	1
6	46	8	2	0
7	38	7	1	0
8	33	2	2	1
9	27	5	7	0
Total	256	51	25	7

**Fig. 4:**
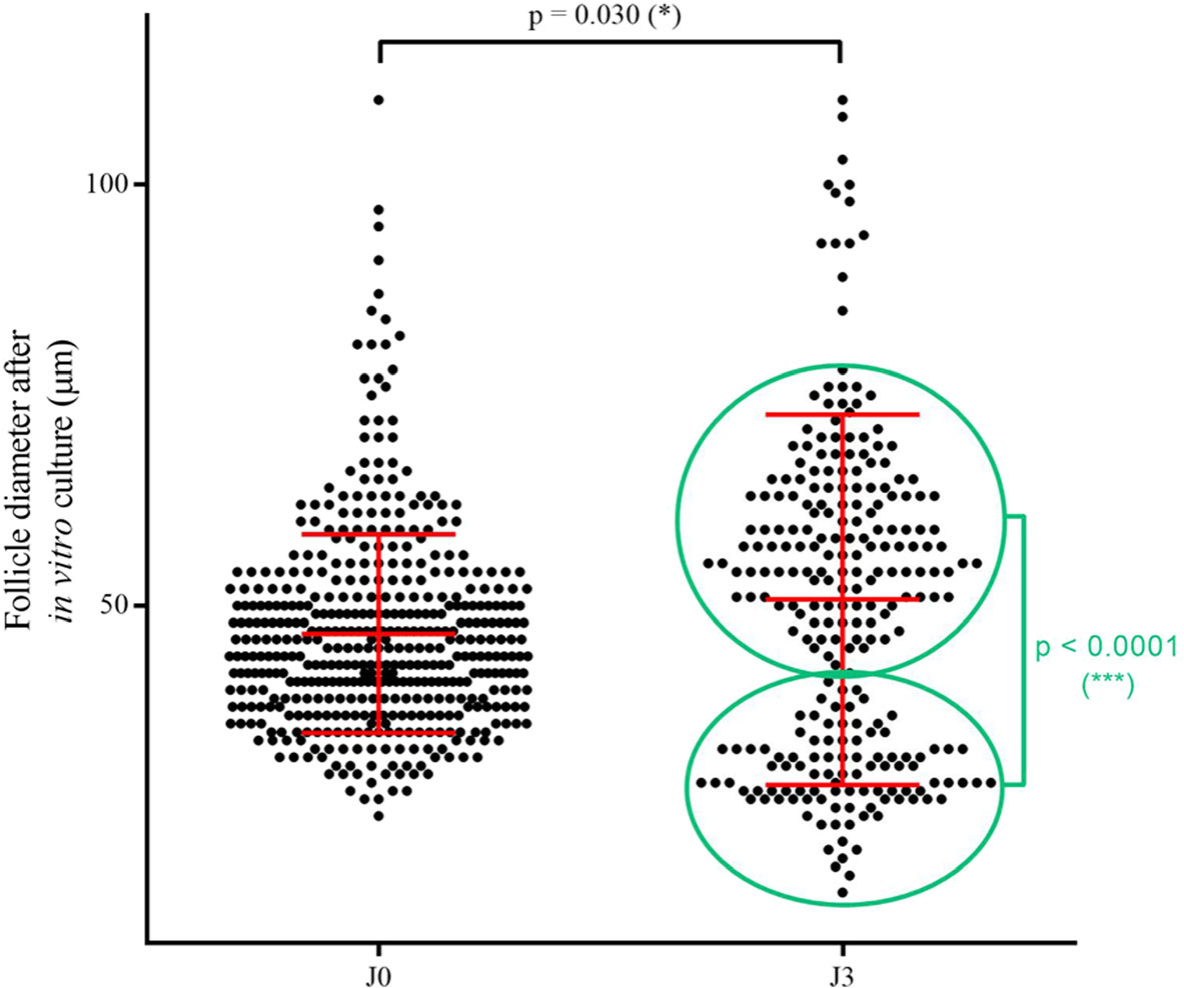
Follicle diameter before and after 3 days of short-term in vitro culture in a fibrin gel. Dots represent each embedded follicles measured. * = statistically significant

In order to evaluate the follicular growth, all the follicles cultured were analyzed right after embedding them in fibrin gels and 3 days after their in vitro culture. At day 0, the mean diameter of follicles was 47.64 ± 5.99 μm (*n* = 453 follicles in 9 fibrin scaffolds). After 3 days of in vitro culture in a fibrin-based scaffold, the mean diameter of follicles significantly increased and was 55.64 ± 9.89 μm (*n* = 263 follicles in 9 fibrin scaffolds) (*p* = 0.030). At day 3, two significantly different populations of follicles were observed (*p* < 0.0001) (Fig. 4). Indeed, 37.6% (mean = 30.15 ± 5.02 μm; 99/263 follicles) of the isolated follicles had a diameter less than or equal to 42 μm, whereas 62.4% (mean = 63.11 ± 18.78 μm; 164/263 follicles) had a diameter greater than 42 μm (Fig. 4).

### Modelization: addition of leukemic cells in human ovarian cell suspensions and isolation of preantral follicles

**Fig. 5:**
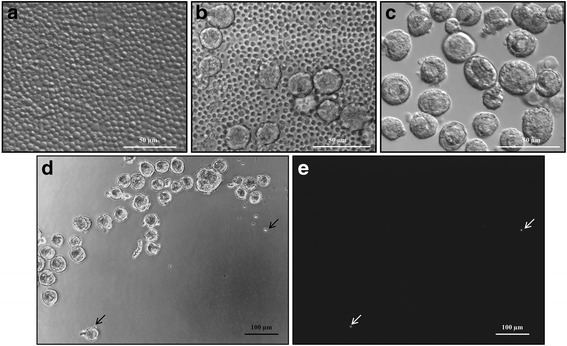
Cell suspensions observed under an inverted micromanipulation microscope. **a** Leukemic cell suspension obtained after thawing, corresponding to positive controls; **b** Ovarian cell suspension, containing follicles and leukemic cells, without wash; **c** Ovarian cell suspension obtained after 3 washes of isolated follicles; **d** An example of ovarian cell suspension obtained after 3 washes of isolated follicles, with two leukemic cells visible (black arrows); **e** The same ovarian cell suspension obtained after 3 washes under inverted fluorescence microscope, showing the presence of two leukemic cells stained by using a fluorescent cell tracer (white arrows)

An excess of leukemic cells was added to the cell suspension obtained after dissociation of ovarian cortical strips by the GMP grade collagenase NB6. We chose to use collagenase NB6 in the following experiments, because its GMP grade makes it usable in the clinic and also because it generates less debris, as previously observed (Fig. 1). A total of 1068 follicles were picked up during leukemic cell addition in healthy ovarian cell suspensions (Fig. 5a-b). Leukemic cells may adhere to isolated follicles, but after three washes only rare leukemic cells were observed under inverted microscope (Fig. 5c). Observation of ovarian follicular suspensions after 3 washes under an inverted fluorescence microscope confirmed that leukemic cells were mostly eliminated by the process (Fig. 5d-e).

**Fig. 6:**
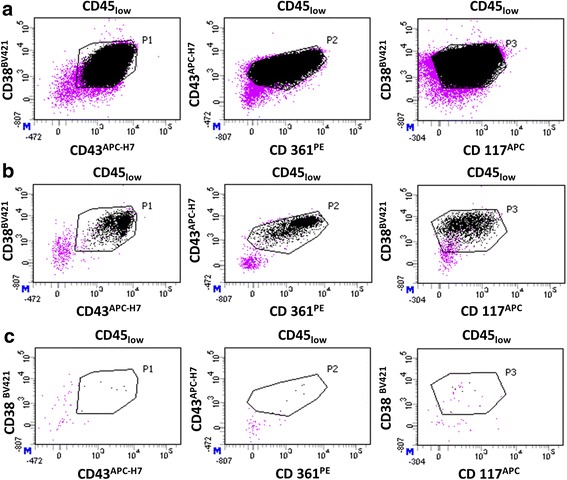
Quantification of leukemic cells by MFC in an ovarian suspension containing isolated follicles. Only viable nucleated cells (SYTO13^+^/7-AAD^−^), which are CD45_*low*_ were represented (purple dots). Black dots represent leukemic cells detected with a typical immunophenotype CD38^+^/CD43^+^/CD361^+^/CD117^+^ among the population of CD45_*low*_ cells. A positive event must be at the intersection of gates P1 (CD38 versus CD43), P2 (CD43 versus CD361), and P3 (CD38 versus CD117). **a** Positive control (leukemic cells); **b** MFC analysis of follicles isolated from healthy ovarian suspension after addition of 10^6^ leukemic cells, before any wash (*n* = 2069 events detected); **c** MFC analysis of isolated follicles after 3 washes (*n* = 7 events detected)

The number of leukemic cells present in ovarian cell suspensions was evaluated by MFC. In the positive control, the cluster of leukemic cells was homogeneous and easily detectable (Fig. 6a). After adding leukemic cells to the ovarian cell suspensions, the number of events detected was low after the first follicle pickup without wash (Fig. 6b), and drastically lower after three washes, even when adding 10^6^ leukemic cells (Fig. 6c).

A total of 19,015 leukemic cells (mean = 792 ± 1402 leukemic cells; min = 1; max = 4889) were detected before any wash, whereas only 61 leukemic cells (mean = 3 ± 5 leukemic cells; min = 0; max = 22) were found after 3 washes, no matter the dilution, across the 24 experiments (*n* = 1068 washed follicles).

Among the 24 experiments, only one cell suspension was positive after 3 washes in IVF medium with 22 events detected, and 23 ovarian cell suspensions were negative (Table 3). When 1 million leukemic cells were added to ovarian cell suspensions, a mean of 2042 ± 1885 events corresponding to leukemic cells were detected before any wash (min = 7; max = 4889; *n* = 8 cell suspensions analyzed), whereas only a mean of 5 ± 7 events were detected after 3 washes (min = 0; max = 22; *n* = 8 cell suspensions analyzed). For 10^5^ leukemic cells added, we found a mean of 316 ± 420 events corresponding to leukemic cells before any wash (min = 1; max = 1265; *n* = 8 cell suspensions analyzed) and a mean of 2 ± 2 events after 3 washes (min = 0; max = 4; *n* = 8 cell suspensions analyzed). Finally, for the lowest concentration of leukemic cells (10^4^), a mean of 19 ± 15 events was detected before any wash (min = 1; max = 37; *n* = 8 cell suspensions analyzed) compared to a mean of 1 ± 1 events after 3 washes (min = 0; max = 2; *n* = 8 cell suspensions analyzed) (Table 4).

**Table 4:** Results obtained after analysis by MFC of the isolated follicle suspensions after adding leukemic cells

					MFC			
					Number of leukemic cells detected		Results	
Patients	Cortical biopsie weight (mg)	Number of isolated follicles	Number of added leukemic cells	Type of leukemia	Before wash	After 3 washes	Before wash	After 3 washes
1	46.2	93	1.10^6^	B-ALL	4889	0	POSITIVE	NEGATIVE
1	46.2	89	1.10^5^	B-ALL	452	4	POSITIVE	NEGATIVE
1	46.2	91	1.10^4^	B-ALL	32	0	POSITIVE	NEGATIVE
2	69.6	4	1.10^6^	B-ALL	7	0	NEGATIVE	NEGATIVE
2	69.6	10	1.10^5^	B-ALL	6	0	NEGATIVE	NEGATIVE
2	69.6	17	1.10^4^	B-ALL	1	0	NEGATIVE	NEGATIVE
3	77	50	1.10^5^	AML	15	3	NEGATIVE	NEGATIVE
3	77	35	1.10^4^	AML	1	0	NEGATIVE	NEGATIVE
3	77	31	1.10^3^	AML	3	0	NEGATIVE	NEGATIVE
4	70.9	11	1.10^6^	AML	177	4	POSITIVE	NEGATIVE
4	70.9	5	1.10^5^	AML	11	1	NEGATIVE	NEGATIVE
4	70.9	3	1.10^4^	AML	3	0	NEGATIVE	NEGATIVE
5	31.7	47	1.10^6^	AML	3434	22	POSITIVE	POSITIVE
5	31.7	46	1.10^5^	AML	388	3	POSITIVE	NEGATIVE
5	31.7	36	1.10^4^	AML	37	2	POSITIVE	NEGATIVE
6	59.3	88	1.10^6^	AML	3799	6	POSITIVE	NEGATIVE
6	59.3	96	1.10^5^	AML	1265	3	POSITIVE	NEGATIVE
6	59.3	65	1.10^4^	AML	36	1	POSITIVE	NEGATIVE
7	38.6	12	1.10^6^	AML	2069	7	POSITIVE	NEGATIVE
7	38.6	20	1.10^5^	AML	206	2	POSITIVE	NEGATIVE
7	38.6	16	1.10^4^	AML	25	2	POSITIVE	NEGATIVE
8	38.6	63	1.10^6^	AML	1942	1	POSITIVE	NEGATIVE
8	38.6	68	1.10^5^	AML	202	0	POSITIVE	NEGATIVE
8	38.6	72	1.10^4^	AML	15	0	NEGATIVE	NEGATIVE
Total		1068			19,015	61		
Mean					792	3		
Standard deviation					1402	5		
Number of experiments	24							

Results obtained before and after three washes of 24 ovarian suspensions containing isolated follicles, in which serial dilutions of leukemic cells (from 10^3^ to 10^6^) were artificially added

In order to see the impact of follicle pickup and washes on follicle survival, we evaluated the viability of 323 isolated follicles after three washes according to the classification V1 to V4, using the calcein AM/ethidium homodimer-1 viability assay. The viability of isolated follicles after undergoing three washes was 97.2% (*n* = 314/323) (Table 5).

**Table 5:** Viability of isolated follicles after three washes

Number of experiments	V1	V2	V3	V4
1	21	10	2	0
2	16	7	3	0
3	20	15	0	0
4	60	8	3	0
5	52	5	1	0
6	92	8	0	0
Total	261	53	9	0

*n* = 6 ovarian strips

## Discussion

The lifespan of women suffering from cancer continues to increase thanks to the curative treatments currently available. However, it is also well-known that cancer treatments are often associated with decreased quality of life, for instance sexual dysfunction [56, 57] and psychosocial disease [58, 59], such as anxiety and depression [60]. Side effects following the administration of therapies may vary according to the type of disease, the type and duration of the treatment, the age of patients and their socio-economic situation. When the administration of highly gonadotoxic treatments is necessary, techniques such as oocyte-, embryo- and ovarian tissue cryopreservation are currently available [7]. For some pathologies including endometrial cancer and ovarian cancer, conservative surgery treatments can also be recommended as fertility sparing strategies [61, 62]. It is important to point out that appropriate fertility preservation counseling is primordial in order to offer adequate fertility preservation techniques and to help women overcome this hardship, but is also mandatory in some countries such as France [63]. Fertility preservation counseling is even more important when ovarian tissue transplantation is associated with a high risk of cancer reseeding, and women should be aware when the autograft of their cryopreserved ovarian tissue would be too dangerous.

When the risk of reseeding malignant cells via the ovarian graft is too high, the transplantation of isolated preantral follicles may be one of the best feasible alternatives. Protocols used for the isolation of preantral follicles can have an impact on their survival, function and morphological integrity [46, 64]. Our study is the first to validate the efficiency of the purified GMP grade collagenase NB6 to successfully isolate a significant number of viable human ovarian follicles.

No significant differences were found between Liberase DH, collagenase IA and the GMP grade collagenase NB6 in terms of yield. However, ovarian suspensions obtained after using GMP grade collagenase NB6 and Liberase DH were less contaminated with undesirable components such as dead cell fragments and conjunctive debris. These components, still present if the digestion is not complete, can stick to isolated follicles, tending to make the follicle isolation process longer and more difficult. The possibility that some follicles observed under the stereomicroscope were actually created by their association with debris and thus creating a heap of extracellular matrix components cannot be excluded in this case.

The mean diameter of follicles isolated with collagenase NB6 was close to results obtained in a study comparing Liberase DH and collagenase IA [43]. Diameters of isolated follicles in our study were significantly lower with collagenases IA and NB6 in comparison to Liberase DH. This might be explained by the additional filtration step used in our procedure to collect follicles of less than 60 μm after the dissociation, contrary to the protocol used for Liberase DH.

The survival rate of isolated follicles was very high in all cases after the dissociation technique, which is in agreement with other studies aiming to isolate human preantral follicles [42, 43, 46, 65].

Ki67 analysis showed that the majority of isolated follicles analyzed after using collagenase NB6 contained Ki67-negative granulosa cells. Even after 3 days of in vitro culture, less than 50% of follicles were non-growing. This shows that most of the isolated follicles were not activated following dissociation with the GMP grade collagenase NB6. Growing follicles (Ki67-positive) were mostly secondary follicles, probably due to their initial development before the dissociation step. The GMP grade collagenase NB6 should not activate the growth of preantral follicles and therefore does not alter their initial condition before their transplantation in leukemia patients.

Indeed we did not observe a high growth of human isolated follicles as seen in other studies performed for 7 days [53, 66, 67], but the time required to initiate their growth must be longer to allow them to fully benefit from the nutrients present in their culture environment. After only 3 days of in vitro culture, we started to observe a heterogeneous distribution of isolated follicles, reflecting the presence of two distinct populations.

According to our results concerning the proliferation of cultured follicles, we can hypothesize that the population of isolated follicles with a diameter below 42 μm must be non-growing follicles still quiescent in the fibrin matrix; whereas the second population with a higher diameter may correspond to activated follicles that have started their development. A longer culture duration of 7 days or more should be done to check this hypothesis and see if more growing follicles (Ki67-positive) would be found.

Small isolated follicles, mostly primordial and primary follicles obtained after dissociation of thawed human ovarian cortical pieces are fragile, so they can rapidly deteriorate in culture even if a large number are viable soon after their isolation [64]. After 3 days of in vitro culture, isolated follicles succeeded in maintaining a high viability, of more than 90%, supporting the idea that the use of GMP grade collagenase NB6 is safe for the isolation of human ovarian preantral follicles.

The fibrin formulation previously used by Paulini et al. [53] was sufficient to produce a scaffold able to support and maintain the 3D structure of human follicles up to 3 days in vitro. However, after this period, we observed a degradation of the matrix around some follicles and a loss of follicles (data not shown). It has already been described in the literature that fibrin clots tend to shrink and degrade after in vitro culture due to fibrinolysis induced by the production of plasminogen activators and connective tissue growth factors by granulosa cells [68–70]. To inhibit this process, other components like aprotinin have been added to the culture media [71]. However, the aim of our study was not to perform an in vitro folliculogenesis but to evaluate the effect of a new GMP grade collagenase on human isolated follicles. This is why we did not extend the culture longer than 3 days, especially as this period was sufficient to evaluate the harmlessness of the GMP grade collagenase NB6.

For clinical purposes, in cases of cancer where the risk of malignant cell contamination is high, it is now clear that new protocols aiming at “isolating human ovarian follicles should include an additional step to confirm the absence of malignant cells in the follicular suspension” as suggested by Amorim and Shikanov [72]. After the validation of our isolation technique, we wanted to ensure the safety of the procedure by adding frozen/thawed malignant cells obtained from AML and ALL patients in healthy ovarian cell suspensions. The possibility of purging the entire ovarian suspensions using a fluorescent labelling system with antibodies specific to malignant cells has been investigated [73]. However, even though it is efficient, this method can be time-consuming and for now these devices cannot be sterilized for re-use in clinics. Soares et al. have demonstrated the safety of their isolation process after the dissociation of artificially contaminated human ovarian tissue from healthy women [42] and from leukemia patients [74], by using RT-qPCR and fluorescence microscopy to detect the presence of leukemic cells in ovarian suspensions. Contrary to their study, we used the technique of MFC, which we have previously codified, as a detection technique to assess the number of living leukemic cells present in ovarian suspensions obtained after the dissociation of human cryopreserved cortical strips [17].

Even though the theoretical number of leukemic cells potentially present in a fragment has been estimated based on biopsy volume and average volume density of blood vessels in the ovarian cortex [42, 75], we cannot accurately state how many cancer cells would be present in cryopreserved ovarian tissue from leukemia patients. To ensure that we were able to eliminate leukemic cells in any case, we decided to add an excess of 10^3^ to 10^6^ leukemic cells in healthy ovarian suspensions containing isolated follicles. The quantity of malignant cells present in ovarian tissue from leukemia patients would most likely be lower than the number of leukemic cells added in the present study, especially as leukemia patients are likely to have already initiated chemotherapy before cryopreservation [11, 17, 76]. However, even though chemotherapy can decrease the presence of leukemic cells, it has been shown that it cannot completely eliminate the risk of contamination [12, 15, 49].

During the isolation procedure, before any wash, it can be difficult to distinguish leukemic cells from stromal cells. Indeed, as observed by Soares et al., the size of leukemic cells is close to that of stromal cells [42]. Moreover, the diameter of the denudation pipette used for all experiments was large enough to allow the aspiration of debris and leukemic cells during the follicle pickup.

In most of our experiments, the number of leukemic cells present in the ovarian cell suspensions after three washes would not be sufficient to potentially induce a relapse of the disease, as according to the literature, the level of minimal residual disease in blood and bone marrow is considered positive when more than 20 positive events are detected by MFC [50]. In only one case in our study, the number of leukemic cells detected was higher than 20 events after three washes. This case corresponded to a suspension in which 1 million leukemic cells were added; knowing that such a high amount of malignant cells would likely not be found in cryopreserved ovarian biopsies from leukemia survivors, these results are quite promising. The viability of isolated follicles was not altered by washes, thereby confirming the harmlessness of the isolation technique.

The major advantage of the MFC strategy is to quantify leukemic cells specifically by differentiating them from ovarian cells and normal lymphocytes that could be present in the ovarian cortex. Nevertheless, it is possible that a few leukemic cells could be undetected by MFC if they are aggregated to isolated follicles. Three washes of the isolated follicles into droplets of fresh in vitro fertilization medium were able to reduce the amount of leukemic cells that could potentially contaminate the ovarian suspension. These results also confirmed the efficiency of MFC to detect leukemic cells among a suspension containing washed human isolated follicles. We would like to insist on the fact that, when molecular markers are not available, MFC is the only technique applicable to quantifying the risk of ovarian residual disease, and so it should be offered to all patients with a risk of leukemia reseeding in the future.

## Conclusion

As a conclusion, we succeeded in codifying a new isolation protocol that allows us to obtain a high number of viable follicles from human ovarian cortex using the highly-purified blend enzyme collagenase NB6 produced in accordance with good manufacturing practices. Indeed, the primordial follicles were maintained in their basement statement. In the majority of cases, three washes of the isolated follicles were sufficient to ensure the subsequent carcinologic safety of the final ovarian suspension. This GMP clinical-grade enzyme, in combination with the present isolation technique, will enable the use of isolated human preantral follicles for future clinical applications.

## Abbreviations

ALL: Acute lymphoblastic leukemia
AML: Acute myeloid leukemia
BM: Bone marrow
DMSO: Dimethyl sulfoxide
FSC: Forward light scatter
GMP: Good manufacturing practices
ICSI: Intra cytoplasmic sperm injection
ITS: Insulin, transferring, sodium, selenite
IVF: In vitro fertilization
LAIP: Leukemia-associated immunophenotype
MFC: Multicolor flow cytometry
MRD: Minimal residual disease
PBS: Phosphate-buffered saline
RPMI: Roswell park memorial institute
RT-qPCR: Real time quantitative polymerase chain reaction
SSC: Side light scatter
αMEM: alpha-Minimum essential medium
